# Patients with heart failure with preserved ejection fraction and low levels of natriuretic peptides

**DOI:** 10.1007/s12471-016-0816-8

**Published:** 2016-03-03

**Authors:** W. C. Meijers, T. Hoekstra, T. Jaarsma, D. J. van Veldhuisen, R. A. de Boer

**Affiliations:** Department of Cardiology, University Medical Center, University of Groningen, Groningen, The Netherlands; Faculty of health sciences, Linköping University, Linköping, Sweden

**Keywords:** Heart failure, Preserved ejection fraction, Quality of life, B-type Natriuretic peptide, Biomarkers, Symptoms

## Abstract

**Aims:**

Heart failure with preserved ejection fraction (HFpEF) is common and its management remains difficult. B-type natriuretic peptide (BNP) levels are used to diagnose heart failure, and as an entry criterion for inclusion into trials. We investigated a population of HFpEF patients who had been randomised into a study based on clinical parameters, and compared those with low BNP levels to those with elevated BNP levels.

**Methods:**

We examined patients who had been enrolled in the Coordinating study evaluating Outcomes of Advising and Counselling in Heart Failure (COACH), with preserved left ventricular ejection fraction (LVEF ≥ 40 %), and compared those with low BNP (< 100 pg/ml; *n* = 30) to those with elevated BNP (≥ 100 pg/ml; *n* = 127). Baseline characteristics, comorbidities, biomarkers, quality of life, and outcome parameters (hospitalisations and death) were compared between the groups. To validate our findings, we repeated all analyses for NT-proBNP (< 300 pg/ml and ≥ 300 pg/ml).

**Results:**

Patients were similar with regard to most clinical characteristics (including age, sex, and LVEF), biomarkers, and comorbidities. In contrast, patients with a low BNP had higher body mass index levels (31 kg/m^2^ vs. 27 kg/m^2^; *p* < 0.01) and lower cardiac troponin I (9 pg/ml vs. 15 pg/ml; *p* = 0.02). In addition, these patients were less frequently prescribed diuretics and beta-blockers. No differences in quality of life, heart failure related symptoms and the primary and secondary outcomes were observed between these groups. These observations were confirmed for NT-proBNP.

**Conclusion:**

Among the patients with clinically diagnosed HFpEF, those with low BNP are strikingly similar to those with elevated BNP levels, except for BMI, which was significantly higher in these patients.

**Electronic supplementary material:**

The online version of this article (doi:10.1007/s12471-016-0816-8) contains supplementary material, which is available to authorized users.

## Introduction

Heart failure with preserved ejection fraction (HFpEF) is a prevalent cause of cardiovascular morbidity and mortality [1] and the incidence is still increasing [2]. Patients with either HFpEF or heart failure with reduced ejection fraction (HFrEF) are generally comparable regarding signs, symptoms and quality of life (QoL) [3]. But HFpEF patients are more often elderly, female and more frequently have hypertension, atrial fibrillation and other comorbidities [4], whereas HFrEF patients have a higher prevalence of coronary artery disease and myocardial infarction [5]. Due to the presence of these comorbidities, often with heart failure-like symptoms, the diagnosis of HFpEF is difficult, and in fact some patients with assumed HFpEF might not have heart failure, but suffer from other conditions such as anaemia, lung disease, or depression.

The guidelines of the European Society of Cardiology (ESC) state that for a diagnosis of heart failure, untreated patients with symptoms of heart failure should have at least B-type natriuretic peptide (BNP) levels of 100 pg/ml to confirm a possible diagnosis of heart failure (or NT-proBNP levels of 300 pg/ml) [6]. In a very recent meta-analysis [7], 37 unique study cohorts with over 15,000 test results were available, and the proposed rule-out threshold for BNP recommended by the 2012 ESC guidelines was shown to have excellent ability to exclude acute heart failure. However, no distinction could be made between HFpEF and HFrEF patients as data on BNP cut-off points in HFpEF are rare and ill-validated. Interestingly, a group of HFpEF patients with BNP levels below 100 do not officially meet the diagnostic criteria. This raises the question whether and how these patients, who are a clinical reality, differ from patients with HFpEF and BNP levels ≥ 100 pg/ml.

Therefore, we compared HFpEF patients with or without low BNP levels for their baseline characteristics, heart failure symptoms, biomarkers, QoL measurements and outcome parameters.

## Methods

### Patient population

Data were collected as part of the Coordinating study evaluating Outcomes of Advising and Counselling in Heart failure (COACH), as described in detail elsewhere [8, 9]. In brief, patients who were admitted for heart failure were enrolled in COACH before discharge, and randomised to standard of care or to nurse-led interventions. In the current sub-study only patients with a left ventricular ejection fraction (LVEF) ≥ 40 %, with complete data on BNP and QoL were included, as previously described [10]. The study was performed in accordance with the principles outlined in the Declaration of Helsinki and was approved by the Medical Ethics Committee in each participating centre. All subjects provided informed consent. Further details are described in the Online Supplement.

### Endpoints

The primary outcome was all-cause mortality and/or rehospitalisation for heart failure after 18 months. Secondary outcomes were all-cause mortality, heart failure rehospitalisation, cardiovascular rehospitalisation, or all-cause rehospitalisation after 18 months. We also analysed all-cause mortality after 36 months. An independent endpoint committee adjudicated all endpoints [11].

### Biochemical measurements

An extensive description of the assays used can be found in the Online supplement.

### Quality of life and heart failure symptoms

QoL measurements were collected during hospitalisation and were assessed in two different ways. Global well-being was assessed by Cantril’s Ladder of Life [12] and the Medical Outcome Study 36-item General Health Survey (RAND36) assessed disease generic QoL [13]. Both established measurements are further explained in the Online Supplement together with the assessment of heart failure symptoms.

### Statistical analyses

Descriptive statistics were used to characterise the study population. Cox proportional hazards regression analyses were performed to adjust for the time to event. Kaplan-Meier curves were constructed for the different time to event evaluations. We repeated our analyses for patients with low NT-proBNP and high NT-proBNP levels.

*P*-values below < 0.05 were considered to denote significant differences. Analyses were performed with STATA software (version 13.0, Stata Corp, College Station, Texas, USA).

## Results

### Patient characteristics

**Fig. 1:**
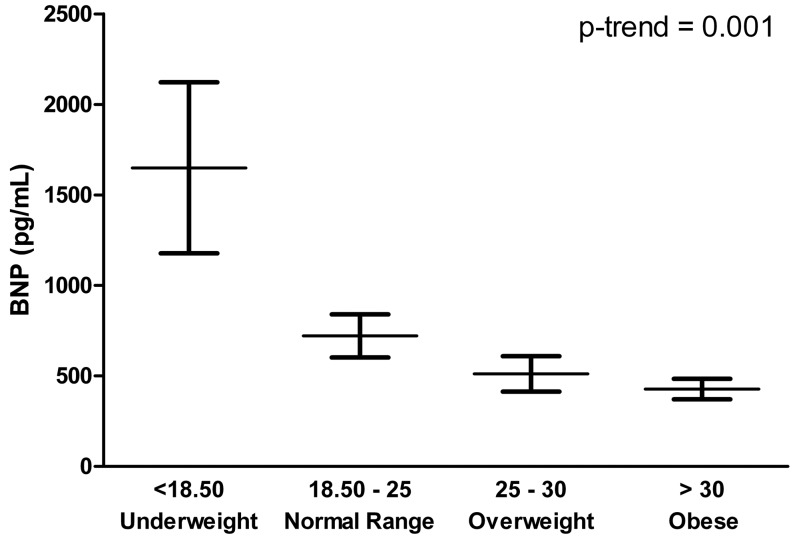
BNP levels stratified by the World Health Organization BMI classification scale in HFpEF patients

Of the 1023 patients enrolled, 157 patients had an LVEF ≥ 40 %, and complete data on BNP and QoL. Patients had a mean age of 73 (± 9), 45 % were female, and they had a mean LVEF of 51 % (± 9 %) and a median BNP level of 352 pg/ml [149–791 pg/ml]. This is substantially lower than in the overall COACH cohort (median BNP 447 pg/ml [195–888 pg/ml]). Of the 157 patients with an LVEF ≥ 40 %, 30 patients (19 %) had BNP levels lower than 100 pg/ml. Patients with low BNP levels had a higher body mass index (BMI; 31 kg/m^2^ vs. 27 kg/m^2^; *p* < 0.01) and were less often treated with beta-blockers and diuretics (30 % vs. 65 %; *p* < 0.001 and 87 % vs. 98 %, respectively, *p* < 0.01) (Table 1). However, other important clinical characteristics were very comparable between the HFpEF patients with low or elevated BNP. Likewise, comorbidities including chronic obstructive pulmonary disease, diabetes and anaemia were equally frequent in patients with either low or high BNP levels. Biomarkers commonly measured in heart failure patients such as cystatin C, galectin-3, interleukin 6, and neutrophil gelatinase-associated lipocalin (NGAL) showed no differences between the two groups, but cardiac troponin I (cTnI) was significantly lower in patients with low BNP levels (9 pg/ml vs. 15 pg/ml; *p* = 0.02). BNP levels gradually decreased stratified by the World Health Organisation BMI classification scale (underweight < 18.5 kg/m^2^; normal range 18.5–25 kg/m^2^; overweight 25–30 kg/m^2^; obese > 30 kg/m^2^) (p-trend < 0.001) (Fig. 1).

**Table 1:** Baseline characteristics of patients with HFpEF enrolled in the COACH study: overall and stratified by BNP levels

Characteristics	Total (*n* = 157)	BNP < 100 pg/ml (*n* = 30)	BNP ≥ 100 pg/ml (*n* = 127)	*p*-value
Age (y), mean (SD)	73 (9)	72 (9)	73 (9)	0.51
Female, *n* (%)	71 (45)	12 (40)	59 (47)	0.52
SBP (mmHg), mean (SD)	125 (22)	125 (25)	125 (22)	0.94
DBP (mmHg), mean (SD)	69 (13)	73 (16)	68 (12)	**0.04**
Heart rate (bpm), mean (SD)	72 (12)	73 (13)	72 (12)	0.51
BMI (kg/m^2^), mean (SD)	28 (6)	31 (7)	27 (5)	< **0.01**
**HF history**				
NYHA class II	88 (56)	12 (40)	76 (60)	0.10
III	61 (39)	15 (50)	46 (36)	
IV	8 (5)	3 (10)	5 (4)	
LVEF (%), mean (SD)	51 (9)	53 (10)	50 (8)	0.06
Previous MI, *n* (%)	51 (33)	6 (20)	45 (35)	0.10
Distance 6MWT, mean (SD)	212 (131)	169 (106)	222 (135)	0.09
**Comorbidities**				
Asthma	6 (4)	0 (0)	6 (5)	0.22
Atrial fibrillation	79 (50)	15 (50)	64 (50)	0.97
Anaemia	43 (43)	6 (33)	37 (45)	0.38
COPD	53 (34)	11 (37)	42 (33)	0.71
Diabetes	45 (29)	8 (27)	37 (29)	0.79
Hypertension	78 (50)	16 (53)	62 (49)	0.66
Stroke	26 (17)	5 (17)	21 (17)	0.99
**Treatment**				
ACEi/ARB, *n* (%)	123 (78)	25 (83)	98 (77)	0.46
β-Blocker, *n* (%)	92 (59)	9 (30)	83 (65)	< **0.001**
Loop diuretic, *n* (%)	151 (96)	26 (87)	125 (98)	< **0.01**
MRA, *n* (%)	67 (43)	11 (37)	56 (44)	0.46
Digoxin, *n* (%)	49 (31)	8 (27)	41 (32)	0.55
**Laboratory measurements**				
Sodium (mmol/l), mean (SD)	139 (4)	138 (6)	139 (4)	0.31
Potassium (mmol/l), mean (SD)	4 (1)	4 (1)	4 (1)	0.44
Urea (mmol/l), mean (SD)	14 (8)	16 (9)	13 (8)	0.11
Creatinine (µmol/l), mean (SD)	127 (61)	129 (51)	126 (63)	0.84
eGFR (ml/min per 1.73 m^2^), mean (SD)	54 (21)	51 (16)	54 (22)	0.42
**Biomarkers**				
BNP (pg/ml), median [IQR]	352 [149–791]	53 [34–72]	457 [244–864]	< **0.001**
Cystatin C (µg/ml), median [IQR]	11.3 [7.8–15.7]	11.2 [9.2–19.0]	11.4 [7.7–15.7]	0.84
Galectin-3 (ng/ml), median [IQR]	19 [15–25]	19 [14–27]	19 [15–25]	0.77
Interleukin 6 (ng/ml), median [IQR]	12 [7–23]	11 [6–22]	12 [7–24]	0.70
NGAL (ng/ml), median [IQR]	113 [87–165]	147 [81–181]	109 [87–147]	0.34
Troponin I (pg/ml), median [IQR]	14 [6–31]	9 [4–13]	15 [7–35]	**0.02**

*BNP* B-type natriuretic peptide, *SBP* systolic blood pressure, *DBP* diastolic blood pressure, *BMI* body mass index, *HF* heart failure, *NYHA* New York Heart Association, *LVEF* left ventricular ejection fraction, *MI* myocardial infarction, *6MWT* 6-minute walk test, *COPD* chronic obstructive pulmonary disease, *ACEi* angiotensin-converting-enzyme inhibitor, *ARB* angiotensin II receptor blocker, *MRA* mineralocorticoid receptor antagonist, *eGFR* estimated glomerular filtration rate, *NGAL* neutrophil gelatinase-associated lipocalin.

### Quality of life

The mean score on global well-being, as measured with Cantril’s Ladder of Life, did not differ significantly between HFpEF patients with low BNP levels and high BNP levels (Table 2). Also, no significant differences were observed between the two groups in any of the dimensions of the RAND36 (Table 2).

**Table 2:** Quality of life and symptoms of patients with HFpEF enrolled in the COACH study: overall and stratified by BNP levels

Characteristics	Total (*n* = 157)	BNP < 100 pg/ml (*n* = 30)	BNP ≥ 100 pg/ml (*n* = 127)	*p*-value
**Ladder of Life**				
Well-being	6 (2)	6 (2)	6 (2)	0.60
**RAND-36**				
Physical functioning, mean (SD)	33 (25)	27 (21)	34 (26)	0.15
Social functioning, mean (SD)	58 (31)	61 (32)	57 (31)	0.51
Role limitation physical, mean (SD)	18 (33)	18 (32)	18 (33)	0.93
Role limitation emotional, mean (SD)	52 (46)	51 (47)	53 (46)	0.86
Mental health, mean (SD)	67 (21)	63 (21)	67 (21)	0.32
Bodily pain, mean (SD)	62 (34)	59 (35)	63 (33)	0.49
General health, mean (SD)	43 (18)	38 (19)	44 (18)	0.10
Health change, mean (SD)	27 (23)	26 (20)	27 (24)	0.84
**Symptoms**				
Oedema, *n* (%)	108 (69 %)	25 (83 %)	83 (65 %)	0.06
Sleep disturbance, *n* (%)	105 (67 %)	18 (60 %)	87 (69 %)	0.37
Fatigue, *n* (%)	138 (88 %)	26 (87 %)	112 (88 %)	0.82
Dyspnoea, *n* (%)	150 (96 %)	30 (100 %)	120 (95 %)	0.19
Cough, *n* (%)	101 (64 %)	17 (57 %)	84 (66 %)	0.33
Loss of appetite, *n* (%)	76 (48 %)	10 (33 %)	66 (52 %)	0.07
Total number of symptoms (0–6)	4.3 (1.3)	4.2 (1.3)	4.3 (1.2)	0.57

*BNP* B-type natriuretic peptide.

### Symptoms of heart failure

HFpEF patients in the COACH study were very symptomatic: patients reported on average four symptoms of heart failure. The most reported symptoms of heart failure were dyspnoea (96 %) and fatigue (88 %). Patients with low BNP levels did not differ in reported symptoms from patients with high BNP levels (Table 2).

### Low BNP and predictive value

**Fig. 2:**
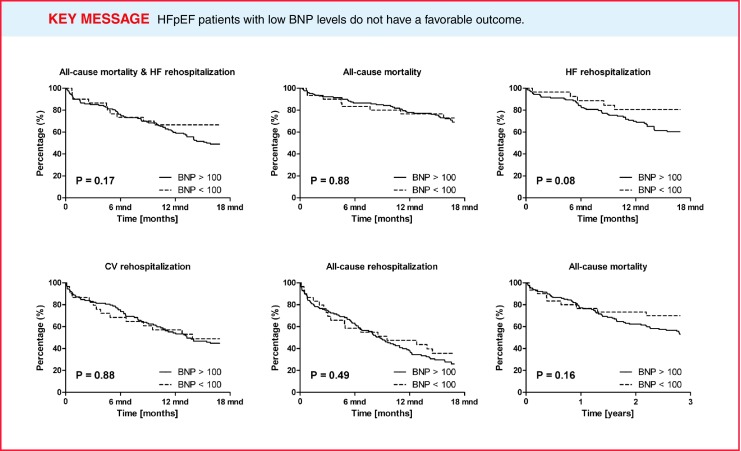
Kaplan-Meier curves for various outcome parameters in patients with HFpEF, stratified by BNP level

In the unadjusted Cox proportional hazard analyses no significant prediction was observed for patients with low BNP levels regarding various outcome parameters (Table 3). Kaplan-Meier curves and the log-rank test on all the outcomes showed no significant differences when patients were stratified according to BNP levels (Fig. 2).

### NT-proBNP

All analyses were also performed using NT-proBNP levels below and above 300 pg/ml; similar observations were made as with BNP. Results are presented in Supplementary Tables 1, 2 and 3 and Supplementary Fig. 1.

**Table 3:** Cox proportional hazard analyses for different endpoints, comparing subjects with low BNP vs. high BNP.

Endpoint	Hazard ratio	95 % CI	*P*-value
**18 months**			
All-cause mortality & HF rehospitalisation	0.63	0.33–1.23	0.179
All-cause mortality	0.94	0.44–2.02	0.877
HF rehospitalisation	0.45	0.18–1.14	0.091
CV rehospitalisation	0.96	0.54–1.71	0.883
All-cause rehospitalisation	0.53	0.24–1.17	0.117
**36 months**			
All-cause mortality	0.84	0.50–1.39	0.493

*HF* heart failure, *CV* cardiovascular.

## Discussion

The main finding of our study is that a group of patients exists who present and are admitted with heart failure symptoms and who, according to the current ESC guidelines, are not likely to be diagnosed with HFpEF because of a too low BNP level. These patients suffer at least as much from their condition as patients who do meet the diagnostic criteria of HFpEF. Further, they do not substantially differ from patients with HFpEF and BNP levels ≥ 100 pg/ml on a broad range of characteristics and heart failure symptoms. Most strikingly, the major difference was the higher BMI levels. Regarding different outcome parameters, we observed no differences between patients with low or high BNP levels, although we had limited power to ascertain this.

### HFpEF and BNP levels

Although there is growing interest in HFpEF, there is limited understanding about the pathology of HFpEF. Natriuretic peptide levels have been advocated to aid the diagnosis of HFpEF. But our findings are in concert with emerging literature that natriuretic peptide testing in HFpEF is not straightforward. Yamamoto et al. have already stated that we should be cautious in using BNP alone in the diagnostic work-up, because BNP concentrations increase in normal, healthy older and/or female individuals, and in those with renal dysfunction and atrial fibrillation, and decrease in obese subjects [14].

So although BNP levels have powerful prognostic value, the diagnostic value of this biomarker is less clear. The ESC Heart Failure guidelines of 2008 introduced a requirement of plasma BNP levels ≥ 100 pg/ml for diagnosing heart failure, in addition to the presence of symptoms of heart failure. As a result, patients with heart failure symptoms but with BNP levels < 100 pg/ml do not ‘officially’ have a diagnosis of heart failure, and physicians are advised to actively seek an alternative diagnosis. Therefore, it is expected that the prevalence of comorbidities among this patient population will be higher than in patients with high BNP levels. A recent paper by Paulus et al. hypothesises that comorbidities may actually drive the myocardial dysfunction in HFpEF [15]. However, in our study we cannot clearly establish such a gradient between BNP levels and the number of comorbidities. The one exception was BMI. It has been published by others [16, 17] that BMI is a strong confounder of natriuretic peptides. Therefore, patients with a high BMI and low BNP levels may have ‘concealed’ heart failure, with disproportionately low BNP levels not properly reflecting left ventricular wall stress.

Usually, BNP directly reflects left ventricular wall stress, but apparently BMI interferes with this relationship. The one finding that validates the notion that lower BNP really associates with lower stress to the heart is our observation that also cTnI was lower in patients with lower BNP. Cardiac troponins are increasingly recognised as a major prognostic factor in heart failure [18]. Whether obesity has a relationship with cTnI levels in acute heart failure remains unknown.

### BNP cut-off point in HFpEF

In the Breathing Not Properly (BNP) Multinational Study, renal function correlated weakly with BNP levels, but more importantly it influenced the optimal BNP cut-off point [19]. Therefore, the ESC working group recommended an alternative cut-off point of 200–250 pg/ml to be considered in these patients [20].

Obesity also impacts BNP levels, even in subjects without heart failure. In the Framingham Study, multivariable-adjusted mean plasma BNP levels in lean (< 25 kg/m^2^), overweight (25–29.9 kg/m^2^), and obese (≥ 30 kg/m^2^) subjects were 21.4, 15.5 and 12.7 pg/ml, respectively [21]. Not only obesity but also diabetes was associated with lower plasma BNP levels [21]. Ideally, these clinical aspects need to be taken into account when assessing a patient’s BNP level; however the attending clinician clearly favours a single cut-off point.

Van Veldhuisen et al. [22] reported that although the BNP levels are lower in HFpEF patients compared with HFrEF patients, the prognosis in both patient groups is comparable given a certain BNP value. These findings were recently strengthened by the same observation by Kang et al. [23]. Whether natriuretic peptides are the best biomarkers to predict outcome in the low range is less well studied. Meijers et al. recently investigated whether and what biomarkers could assess low risk in patients with heart failure. Low levels of BNP or NT-proBNP did not strongly predict outcomes in these patients, whereas other biomarkers performed better and identified patients with a low risk for an adverse outcome [24].

### Clinical implementation: HFpEF with low versus high BNP

Our results show no apparent differences between the two patient groups in the frequency of comorbidities, except for BMI levels. As mentioned above, BNP levels decrease in obese patients [21]; thus, these patients may still suffer from HFpEF despite their (pseudo) lower BNP levels. These observations of the current study raise important questions regarding the use and interpretation of BNP levels as a biomarker for HFpEF.

The exact condition of ‘HFpEF’ patients with low BNP levels remains unclear, resulting in a dilemma for clinical practice when it comes to how to work up and how to treat these patients. Recently it was demonstrated that comorbidities might also influence the response to BNP-guided therapy [25]. Our data suggest that the clinical work-up should probably be identical, as the clinical risk factor, the physical and mental unwell-being and the outcomes are almost identical. In particular, similar to patients with ‘real’ HFpEF, their physical functioning is low, and regardless of possible pharmacological treatment, these patients may likely benefit from exercise treatment, and referral to a rehabilitation centre would be advised [26]. Weight loss may paradoxically lead to an increase in BNP levels but a better performance score.

## Strengths and limitations

This post-hoc study has several limitations. We defined HFpEF as an LVEF ≥ 40 % [27, 28], realising that the optimal LVEF cut-off point is a matter of debate. The COACH study was performed before the era when echocardiography was a necessity for heart failure diagnostics and therefore the echocardiography data presented in this study cannot provide exact phenotyping of HFpEF patients. It could be suggested to measure BNP levels serially and use changes in BNP levels over time, instead of a single measurement, to diagnose HFpEF; unfortunately we could not address this issue. The sample size was small and these data should be regarded as hypothesis generating. However, due to the scarce data of HFpEF patients with low and high BNP, and as far as we are informed, our data are the first to address this subpopulation. Further, using our sample we were able to demonstrate significant differences which appear biologically plausible, especially the difference in BMI.

Another strength of the study is that we enrolled real-life patients presenting with dyspnoea in specialised heart failure centres.

## Conclusion

Patients with heart failure and a preserved ejection fraction and plasma BNP levels < 100 pg/ml have the same clinical characteristics, an equal number and frequency of comorbidities, equally severe heart failure symptoms with impaired QoL, and the same poor outcome, when compared with patients with heart failure and a preserved ejection fraction and BNP levels ≥ 100 pg/ml. The major difference between the two groups was a higher BMI in HFpEF patients with low BNP. It should be considered to evaluate and treat patients with suspected HFpEF and BNP levels below the ESC guideline threshold in a comparable manner to that used for ‘official’ HFpEF patients.

### Sources of funding

This work was supported by an Innovational Research Incentives Scheme program of the Netherlands Organization for Scientific Research (VIDI grant 917.13.350), to RAdB and by the Netherlands Heart Foundation (grant 2015T034) to WCM. The COACH trial was supported by grant 2000Z003 from the Netherlands Heart Foundation and by additional unrestricted grants from Biosite France SAS (Jouy-en-Josas, France), Roche Diagnostics Nederland BV (Venlo, the Netherlands), and Novartis Pharma BV (Arnhem, the Netherlands).

## Electronic supplementary material

(DOCX 229 kb)
