# Supplementary material for: Patients with heart failure with preserved ejection fraction and low levels of natriuretic peptides
Source: Neth Heart J. 2016 Mar 3;24(4):287–95. doi: 10.1007/s12471-016-0816-8 (PMC4796061; doi:10.1007/s12471-016-0816-8)
Supplement: Supplementary file 1 — (DOCX 229 kb) [file 12471_2016_816_MOESM1_ESM.docx]

**Supplementary Material**

**Patient population**

The COACH trial consists of 1023 heart failure patients from 17 hospitals in the Netherlands who were enrolled after hospitalisation for heart failure. The diagnosis was based on a combination of typical signs and symptoms according to the ESC guidelines for which a hospital stay was considered necessary. During hospitalisation all patients received standard care, both pharmacological and non-pharmacological. After baseline measurement, patients were randomised to receive extra education and support or care as usual only.

The study was performed in accordance with the principles outlined in the Declaration of Helsinki and was approved by the Medical Ethics Committee in each participating centre. All subjects provided informed consent.

**Additional laboratory measurements**

Plasma BNP concentrations were measured using a fluorescence immunoassay kit (Triage, Biosite Incorporated, San Diego, California) and the measurable range of BNP assays was 5.0 to 5000 pg/ml. N-terminal pro-brain natriuretic peptide (NT-pro-BNP) was measured by Roche Diagnostics, Mannheim, Germany using the Elecsys proBNP ELISA. Cystatin C and neutrophil gelatinase-associated lipocalin (NGAL) were measured by Alere San Diego, Inc., San Diego, CA, USA, using competitive enzyme-linked immunosorbent assays (ELISAs) on a Luminex® platform. Galectin-3 plasma levels were measured using a commercial enzyme-linked immunosorbent assay (BG Medicine, Waltham, MA) (16),(17). Cardiac troponin I (cTnI) and Interleukin-6 (IL-6) were measured using high-sensitive single molecule counting (SMCTM) technology (RUO, Erenna Immunoassay System, Singulex Inc., Alameda, CA, USA).

**Quality of life and heart failure symptoms**

Global well-being was assessed by Cantril’s Ladder of Life. This is a single-item measure which asks the patient to rate their sense of well being on a ladder, with 10 reflecting the best possible life imaginable and 0 reflecting the worst possible life imaginable. Cantril’s Ladder of Life has been used in various cardiovascular studies and is considered to be a valid measure of global well-being [12]. A higher score indicates better well-being.

Disease generic QoL was assessed by the Medical Outcome Study 36-item General Health Survey (RAND36), a self-report questionnaire of general health status and comparable to the Short-Form-36 Health Survey (SF-36) [13]. The RAND36 is a well-validated generic, 36-item questionnaire that includes nine health concepts that represent dimensions of QoL: physical functioning, social functioning, role limitations because of physical functioning, role limitations because of emotional functioning, mental health, vitality, bodily pain, general health and perceived health change. Each dimension has a score between 0 and 100; a higher score means better health.

Symptoms of heart failure were assessed from an interview comprising ten structured questions. During this interview, patients were asked whether they had experienced the following symptoms during the last month: ankle oedema during the day, ankle oedema when getting out of bed in the morning, sleep disturbance, fatigue, breathlessness at rest and during exertion, orthopnoea, coughing, dry cough or loss of appetite. These ten symptoms were clustered in six symptom indexes: oedema, sleep disturbance, fatigue, dyspnoea, coughing and loss of appetite. A total heart failure symptom score was obtained from the sum of the six symptom indices.

Supplemental Table 1. Baseline characteristics of patients with HFPEF enrolled in the COACH study, overall and stratified by NT-proBNP levels.

| **Characteristics** | **Total**  **(n=110)** | **NT-proBNP < 300 pg/ml**  **(n=11)** | **NT-proBNP ≥ 300 pg/ml**  **(n=99)** | **p-value** |
| --- | --- | --- | --- | --- |
| Age (y), mean (SD) | 74 (10) | 70 (9) | 74 (10) | 0.19 |
| Female, n (%) | 50 (46) | 5 (45) | 45 (45) | 1.00 |
| SBP (mm Hg), mean (SD) | 127 (23) | 128 (19) | 127 (23) | 0.82 |
| DBP (mm Hg), mean (SD) | 70 (14) | 76 (15) | 69 (13) | 0.12 |
| Heart rate (bpm), mean (SD) | 72 (12) | 73 (14) | 72 (11) | 0.87 |
| BMI (kg/m^2^), mean (SD) | 28 (5) | 31 (4) | 27 (5) | **0.04** |
|  |  |  |  |  |
|  |  |  |  |  |
| **HF history** |  |  |  |  |
| NYHA class II | 58 (53) | 6 (55) | 52 (53) | 0.79 |
| III | 48 (43) | 5 (45) | 43 (43) |  |
| IV | 4 (4) | 0 (0) | 4 (4) |  |
| LVEF (%), mean (SD) | 50 (9) | 52 (11) | 50 (9) | 0.63 |
| Previous myocardial infarction, n (%) | 33 (30) | 4 (36) | 29 (29) | 0.63 |
| Duration of admission (days), mean (SD) | 14 (13) | 8 (3) | 15 (13) | 0.10 |
| Previous hospitalisation for HF, n (%) | 43 (39) | 3 (27) | 40 (40) | 0.40 |
| Distance 6MWT, mean (SD) | 220 (133) | 229 (123) | 219 (135) | 0.82 |
|  |  |  |  |  |
| **Comorbidities** |  |  |  |  |
| Asthma | 6 (6) | 0 (0) | 6 (6) | 0.40 |
| Atrial fibrillation | 57 (52) | 5 (45) | 52 (53) | 0.66 |
| Anaemia | 29 (43) | 2 (33) | 27 (44) | 0.63 |
| COPD | 36 (33) | 4 (36) | 32 (32) | 0.79 |
| Diabetes | 33 (30) | 4 (36) | 29 (29) | 0.63 |
| Hypertension | 56 (51) | 7 (64) | 49 (49) | 0.37 |
| Stroke | 16 (15) | 2 (18) | 14 (14) | 0.72 |
|  |  |  |  |  |
| **Treatment** |  |  |  |  |
| ACEi/ARB, n (%) | 85 (77) | 9 (82) | 76 (77) | 0.70 |
| β-blocker, n (%) | 70 (64) | 3 (27) | 67 (68) | **<0.01** |
| Loop diuretic, n (%) | 103 (94) | 8 (73) | 95 (96) | **<0.01** |
| MRA, n(%) | 49 (45) | 4 (36) | 45 (45) | 0.56 |
| Digoxin, n (%) | 36 (33) | 2 (18) | 34 (34) | 0.28 |
|  |  |  |  |  |
| **Laboratory measurements** |  |  |  |  |
| Sodium (mmol/l), mean (SD) | 138 (4) | 138 (6) | 139 (4) | 0.65 |
| Potassium (mmol/l), mean (SD) | 4 (1) | 4 (1) | 4 (1) | 0.98 |
| Urea (mmol/l), mean (SD) | 13 (7) | 12 (5) | 13 (8) | 0.60 |
| Creatinine (µmol/l), mean (SD) | 124 (62) | 114 (39) | 126 (64) | 0.57 |
| eGFR (ml/min per 1.73 m^2^), mean (SD) | 55 (21) | 56 (15) | 55 (22) | 0.83 |
|  |  |  |  |  |
| **Biomarkers** |  |  |  |  |
| NT-proBNP (pg/ml), median [IQR] | 1772 [758-3780] | 155 [122-215] | 1904 [1062-3956] | **<0.001** |
| Cystatin C (µg/ml), median [IQR] | 11366 [8010-15514] | 9645 [8617-11036] | 11521 [7928-15687] | 0.42 |
| Galectin-3 (ng/ml), median [IQR] | 19 [14-26] | 17 [14-19] | 20 [15-26] | 0.05 |
| Interleukin 6 (ng/ml), median [IQR] | 12 [7-24] | 10 [6-12] | 12 [7-24] | 0.20 |
| NGAL (ng/ml), median [IQR] | 116 [87-168] | 116 [74-153] | 114 [89-169] | 0.64 |
| Troponin I (pg/ml), median [IQR] | 14 [6-31] | 9 [3-18] | 14 [6-32] | 0.29 |

Supplemental Table 2. Quality of life and symptoms of patients with HFPEF enrolled in the COACH study, overall and stratified by NT-proBNP levels.

| **Characteristics** | **Total**  **(n=110)** | **NT-proBNP < 300 pg/ml**  **(n=11)** | **NT-proBNP ≥ 300 pg/ml**  **(n=99)** | **p-value** |
| --- | --- | --- | --- | --- |
| **Ladder of Life** |  |  |  |  |
| Well-being | 6 (2) | 6 (1) | 6 (2) | 0.36 |
|  |  |  |  |  |
| **RAND-36** |  |  |  |  |
| Physical functioning, mean (SD) | 33 (26) | 32 (24) | 33 (26) | 0.89 |
| Social functioning, mean (SD) | 57 (32) | 69 (26) | 56 (33) | 0.18 |
| Role limitation physical, mean (SD) | 19 (33) | 32 (39) | 17 (32) | 0.17 |
| Role limitation emotional, mean (SD) | 50 (46) | 67 (42) | 48 (47) | 0.21 |
| Mental health, mean (SD) | 66 (22) | 72 (21) | 66 (22) | 0.38 |
| Bodily pain, mean (SD) | 61 (33) | 55 (35) | 62 (33) | 0.53 |
| General health, mean (SD) | 42 (18) | 36 (15) | 43 (19) | 0.26 |
| Health change, mean (SD) | 25 (21) | 32 (20) | 25 (21) | 0.29 |
|  |  |  |  |  |
| **Symptoms** |  |  |  |  |
| Oedema, n (%) | 78 (71) | 8 (73) | 70 (71) | 0.89 |
| Sleep disturbance, n (%) | 75 (68) | 8 (73) | 67 (68) | 0.73 |
| Fatigue, n (%) | 102 (93) | 11 (100) | 91 (92) | 0.33 |
| Dyspnoea, n (%) | 105 (96) | 11 (100) | 94 (95) | 0.45 |
| Cough, n (%) | 70 (64) | 7 (64) | 63 (64) | 1.00 |
| Loss of appetite, n (%) | 50 (46) | 3 (27) | 47 (47) | 0.20 |
| Total number of symptoms (0-6) | 4.4 (1.2) | 4.4 (1.0) | 4.4 (1.3) | 1.00 |

Supplemental Table 3. Cox proportional hazard analyses for different endpoints, with NT-proBNP levels < 300 pg/ml

| **Endpoint** | **Hazard Ratio** | **95% CI** | **P-value** |
| --- | --- | --- | --- |
| **18 months** |  |  |  |
| All-cause mortality & HF rehospitalisation | 0.28 | 0.07-1.14 | 0.075 |
| All-cause mortality | 0.26 | 0.04-1.90 | 0.184 |
| HF rehospitalisation | 0.40 | 0.10-1.66 | 0.207 |
| CV rehospitalisation | 0.60 | 0.22-1.65 | 0.319 |
| All-cause rehospitalisation | 0.35 | 0.08-1.46 | 0.150 |
| **36 months** |  |  |  |
| All-cause mortality | 0.18 | 0.02-1.28 | 0.087 |

Supplemental Figure 1. Kaplan-Meier curves for various outcome parameters in patients with HFPEF, stratified by NT-proBNP level
